# Psychological distress and uterine fibroids: a bidirectional two-sample mendelian randomization study

**DOI:** 10.1186/s12905-024-03196-8

**Published:** 2024-06-18

**Authors:** Xinyu Han, Tian qiang Wu, Yuanyuan Bian, Lu Chen, Xiaoling Feng

**Affiliations:** 1 Department of First Clinical Medical College, Heilongjiang University of Chinese Medicine, Harbin, China; 2 Department of Gynecology, The First Affiliated Hospital of Heilongjiang University of Chinese Medicine, No. 26, Heping Road, Xiang-fang District, Harbin, Heilongjiang Province China

**Keywords:** Mendelian randomization, UFs, Major depressive disorder, Mood swings, Anxiety or panic attacks, Causality

## Abstract

**Background:**

Observational data indicates a connection between emotional discomfort, such as anxiety and depression, and uterine fibroids (UFs). However, additional investigation is required to establish the causal relationship between them. Hence, we assessed the reciprocal causality between four psychological disorders and UFs utilizing two-sample Mendelian randomization (MR).

**Methods:**

To evaluate the causal relationship between four types of psychological distress (depressive symptoms, severe depression, anxiety or panic attacks, mood swings) and UFs, bidirectional two-sample MR was employed, utilizing single nucleotide polymorphisms (SNPs) associated with these conditions. Both univariate MR (UVMR) and multivariate MR (MVMR) primarily applied inverse variance weighted (IVW) as the method for estimating potential causal effects. Complementary approaches such as MR Egger, weighted median, simple mode, and weighted mode were utilized to validate the findings. To assess the robustness of our MR results, we conducted sensitivity analyses using Cochran’s Q-test and the MR Egger intercept test.

**Results:**

The results of our UVMR analysis suggest that genetic predispositions to depressive symptoms (Odds Ratio [OR] = 1.563, 95% Confidence Interval [CI] = 1.209–2.021, *P* = 0.001) and major depressive disorder (MDD) (OR = 1.176, 95% CI = 1.044–1.324, *P* = 0.007) are associated with an increased risk of UFs. Moreover, the IVW model showed a nominally significant positive correlation between mood swings (OR: 1.578; 95% CI: 1.062–2.345; *P* = 0.024) and UFs risk. However, our analysis did not establish a causal relationship between UFs and the four types of psychological distress. Even after adjusting for confounders like body mass index (BMI), smoking, alcohol consumption, and number of live births in the MVMR, the causal link between MDD and UFs remained significant (OR = 1.217, 95% CI = 1.039–1.425, *P* = 0.015).

**Conclusions:**

Our study presents evidence supporting the causal relationship between genetic susceptibility to MDD and the incidence of UFs. These findings highlight the significance of addressing psychological health issues, particularly depression, in both the prevention and treatment of UFs.

**Supplementary Information:**

The online version contains supplementary material available at 10.1186/s12905-024-03196-8.

## Introduction

Uterine fibroids (UFs), or uterine leiomyomas (UL), represent the most prevalent benign tumors in women of reproductive age. They are primarily characterized by clinical symptoms such as excessive menstrual bleeding, pelvic pain, anemia, and constipation [1]. These fibroids can contribute to various reproductive complications, including impaired fertility, miscarriages, and placental abruption [2]. Moreover, UFs are the leading cause of performing a hysterectomy, accounting for 30–50% of all such procedures. This prevalence exceeds the number of hysterectomies conducted for gynecological cancers [3]. Despite being a benign condition, UFs significantly impact women’s physical and mental health. Consequently, identifying and understanding the risk factors for UFs is crucial for their early prevention and management. Presently, obesity, alcohol consumption, smoking, and a low number of live births are recognized as risk factors for UFs [4–6].

Recently, the link between mental health and UFs has gained increasing attention. A meta-analysis of observational studies revealed a positive correlation between chronic psychological stress and the risk of UFs [7]. A cross-sectional study found a higher incidence of UFs among women who reported more major life events and higher stress intensity [8]. Moreover, a prospective cohort study identified that individuals with high scores on scales measuring symptoms of depression and those who were diagnosed with depression by a healthcare professional had a greater likelihood of developing UFs [9]. Concurrently, cohort studies have indicated that women diagnosed with UF exhibit a higher incidence of depression (Hazard Ratio [HR]: 1.12; 95% CI: 1.10–1.13) and anxiety (HR: 1.12; 95% CI: 1.10–1.13) compared to women without a UF diagnosis [10]. Emotional instability, characterized by frequent and unpredictable fluctuations in one’s emotional state, is not solely a symptom of mental illness but also a prevalent personality trait observed in the general population [11]. Mood swings have been demonstrated to be connected with hypertension, potentially representing another psychological factor contributing to the increased incidence of UFs [12]. Considering that the evidence supporting the correlation between the aforementioned four psychological distress factors and UFs stems from observational studies, inherent limitations exist in addressing confounding factors and reverse causation bias. As a result, the causal relationships within these correlations have not been definitively established.

Mendelian randomization (MR) utilizes natural genetic variation, as identified in genome-wide association studies (GWAS), to simulate randomized controlled trials. This approach employs genetic variation as an instrumental variable, enabling the inference of causal relationships between modifiable exposures and diseases [13]. Alleles are randomly assigned to offspring during the formation of fertilized eggs, resulting in fixed genotypes that remain unaffected by disease progression. This process effectively circumvents confounding bias and reverse causality, thus providing a more reliable basis for establishing causal inferences [14]. MR furnishes a more reliable basis for causal inference, presenting an innovative approach to investigating the etiology-disease relationship. To date, no MR studies have confirmed a bidirectional causal relationship between psychological distress and UFs. Consequently, this article employs both univariate and multivariate MR analysis methods to explore the bidirectional causal relationship between four psychological distress factors—depressive symptoms, major depressive disorder (MDD), anxiety or panic attacks, and mood swings—and UFs.

## Materials and methods

### Study design

**Fig. 1:**
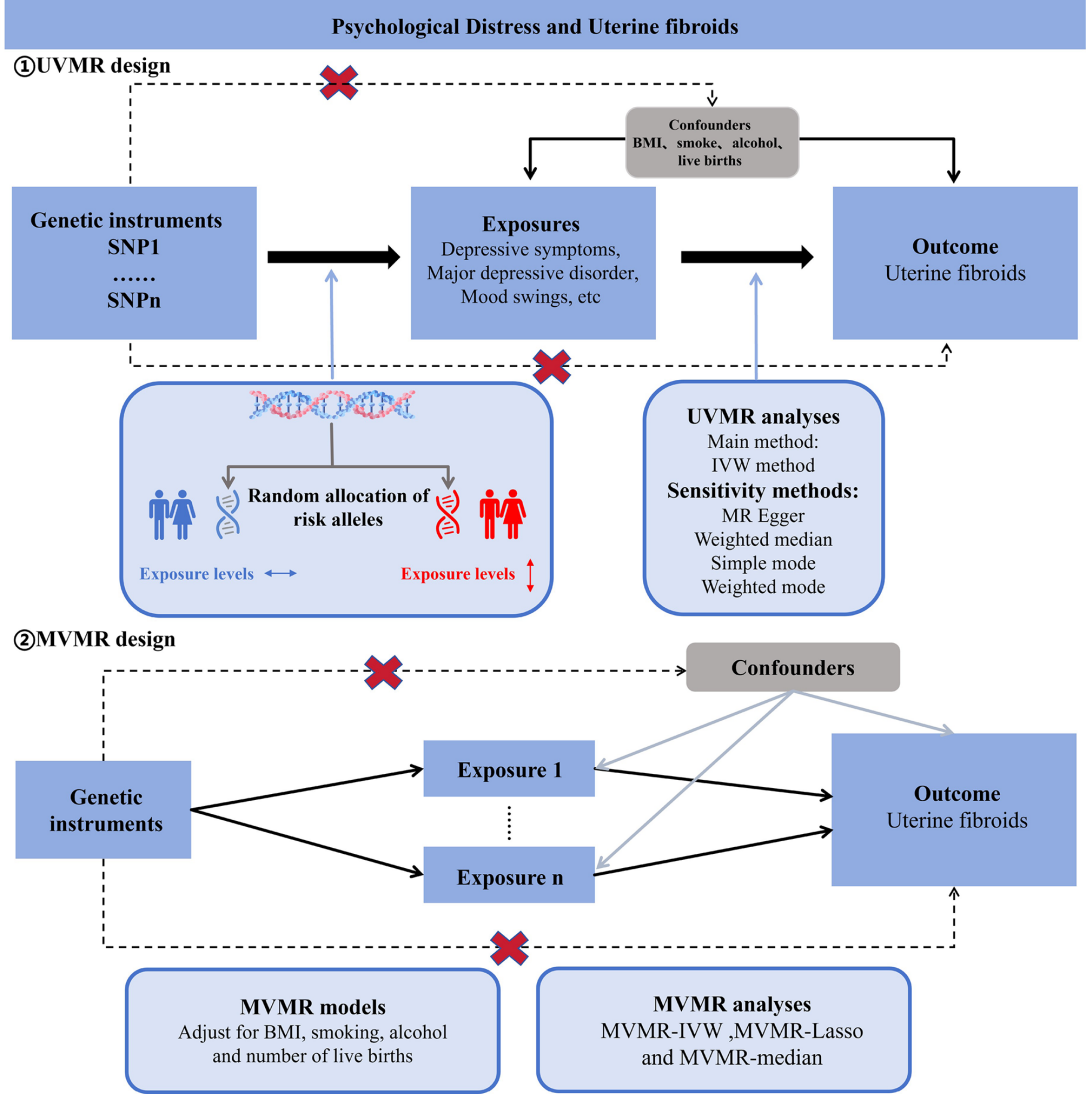
Assumptions and study design of the MR study of the associations between psychological factors and UFs.

A concise depiction of the two-sample MR designs is presented in Fig. 1. We conducted two-sample univariate MR (UVMR) and multivariate MR (MVMR) analyses to thoroughly investigate the associations of four types of psychological distress with UFs. UVMR relies on three primary assumptions: (1) the genetic variants chosen as the instrumental variables (IVs) should be strongly linked to the exposure; (2) the genetic variants should not be correlated with confounding factors; (3) the genetic variants influence the outcome solely through the exposure and not via alternative pathways [15]. The primary premise of MVMR, in contrast to UVMR, pertains to genetic variation related to multiple exposures, while the remaining assumptions align with UVMR [16]. Initially, genetic variables linked to each type of psychological distress were selected to infer the causal relationship with UFs utilizing UVMR. Prior observational clinical trials and MR investigations have presented evidence indicating that body mass index (BMI), smoking, alcohol intake frequency, and number of live births are risk factors for UFs development [4–6]. Consequently, the genetic variations relevant to these four exposures were incorporated into the MVMR model to estimate the direct impact of psychological factors on UFs.

### Data sources

In the present study, the exposures were four types of psychological distress, including depressive symptoms, MDD, anxiety or panic attacks, and mood swings. The research outcome was UFs. We obtained the GWAS summary data of depressive symptoms (*n* = 161,460) from the Social Science Genetic Association Consortium (SSGAC; https://www.thessgac.org/) [17]. The genetic IVs for MDD (170,756 cases and 329,443 controls) were acquired from the Psychiatric Genomics Consortium (PGC; http://www.med.unc.edu/pgc/) [18]. For anxiety or panic attacks (*n* = 337,159; 4,611 cases), we used Neale Lab consortium summary statistics. The database of mood swings was derived from Ben Elsworth’s recently published GWAS study involving 9,851,867 single nucleotide polymorphisms (SNPs). “ https://gwas.mrcieu.ac.uk/datasets/ ”(accessed on April 8, 2024). The summary statistical data for UFs originates from the GWAS study conducted by Sakaue S et al., with a sample size of 258,718 and 24,129,853 SNPs identified [19]. The Neale Lab or MRC-IEU consortium provided combined data on BMI, smoking, alcohol intake frequency, and the number of live births. All individuals involved in the investigation are of European ancestry. Table 1 provides a clear overview of the datasets included in this study.

**Table 1:** Details of studies included in Mendelian randomization (MR) analyses

Traits	Author	GWAS ID	Sample size	Ancestry	Year	Consortium/ PMID
Exposure						
depressive symptoms	Okbay	ieu-a-1000	161,460	European	2016	SSGAC
MDD	Howard DM	ieu-b-102	170,756/329,443	European	2019	PGC
anxiety or panic attacks	Neale	ukb-a-82	4,611/332,548	European	2017	Neale Lab
mood swings	Ben Elsworth	ukb-b-14,180	204,412/247,207	European	2018	MRC-IEU
smoking	Neale	ukb-a-225	33,928/302,096	European	2017	Neale Lab
alcohol intake frequency	Ben Elsworth	ukb-b-5779	462,346	European	2018	MRC-IEU
BMI	Neale	ukb-a-248	336,107	European	2017	Neale Lab
number of live births	Ben Elsworth	ukb-b-1209	250,782	European	2018	MRC-IEU
Outcomes						
UFs	Sakaue S	ebi-a-GCST90018934	21,024/237,694	European	2021	34,594,039

UFs, uterine fibroids; MDD, major depressive disorder; BMI, body mass index

### Selection and evaluation of instrumental variable

We implemented a specific procedure for selecting the IVs to fulfill the three critical assumptions of MR analysis. We extracted SNPs strongly related to MDD, mood swings, and UFs at the *P* < 5 × 10 ^− 8^ significance level. Because of the limited number of available SNPs, a cutoff threshold (*P* < 5 × 10 ^− 6^) was adopted to identify SNPs predictive of depressive symptoms and anxiety or panic attacks. To mitigate the impact of linkage disequilibrium (LD) among the SNPs, we established a stringent criterion (r^2^ < 0.001 and a clumping distance of 10,000 kb), ensuring that the selected IVs were conditionally independent. Only SNPs with the lowest p-values were retained [20]. Furthermore, the potential pleiotropic effects were controlled by extracting the secondary phenotype of each SNP utilizing the LDtrait Tool (https://ldlink.nih.gov/?tab=ldtrait) [21]. As it is widely recognized, factors such as BMI, obesity, alcohol consumption, age at menarche, and number of live births are risk factors for UFs [4–6]. Consequently, we removed any IVs related to these factors or directly connected to UFs. Ultimately, we extracted exposure IVs from the outcome data and conducted data harmonization to exclude SNPs with inconsistent exposure and outcome data alleles.

The robustness of IVs was evaluated employing variance (R^2^) and *F*-statistic to mitigate the influence of weak instrument bias. The formula to calculate the *F*-statistic for each SNP is *F* = R^2^/(1-R^2^)[(N-K-1)/K], where N represents the sample size, K denotes the total number of SNPs selected for MR analysis, and R^2^ reflects the overall proportion of phenotypic differences explained by all the SNPs in our MR model [22]. The R^2^ for each SNP was calculated utilizing the following formula: R^2^ = 2*EAF*(1-EAF)* β^2^, where β is the β coefficient for effect size, and EAF is the effect allele frequency for each SNP [23]. An *F*-statistic exceeding ten was considered significant for the association between IVs and exposure, ensuring weak instrument bias did not affect the results [24].

### Statistical analysis

In order to assess the genetic causal effects, various methodologies, including inverse variance weighted (IVW), MR-Egger, weighted median, weighted mode, and simple mode, were implemented. These methods yielded reliable evidence under different circumstances, with IVW being the primary result [25]. The IVW technique expands the Wald ratio estimator that utilizes meta-analytic principles. It aims to provide an unbiased estimation in an optimal scenario where all the included SNPs are assumed to be legitimate IVs without horizontal pleiotropy or heterogeneity [26]. MR-Egger allows specific SNPs to impact the result by mechanisms other than exposure, providing a dependable and impartial estimation, even when all SNPs are invalid.

Furthermore, the MR-Egger intercept can identify and correct for pleiotropy [25, 27]. Although up to 50% of the data utilized in the study consists of invalid IVs, the weighted median method can still generate dependable estimates of the causal effects [28]. The weighted model method clusters SNPs and computes estimates based on the cluster with the highest number of SNPs [29], which remains valid even if other IVs do not meet the criteria for causal inference in the MR technique [30]. While the simple model method is less effective than IVW, it offers robustness for pleiotropy. Considering previous study findings as a foundation, we accounted for BMI, smoking, alcohol intake frequency, and number of live births in an MVMR analysis [4–6]. This allowed us to estimate the direct impact of psychological distress on UFs independent of risk variables. The techniques we utilized to perform MVMR included IVW and MR-Lasso [29].

In this study, a series of sensitivity analyses were conducted to confirm the stability and reproducibility of the MR results. Cochran’s Q test was employed to evaluate heterogeneity among SNPs, with a p-value exceeding 0.05, which indicated no significant heterogeneity. Additionally, the MR-Egger intercept approach was utilized to estimate the degree of horizontal pleiotropy attributable to IVs. A leave-one-out analysis was also implemented to ascertain whether the MR findings were affected by any specific SNP. Furthermore, the MR-PRESSO method was applied to detect potential outlier SNPs. The p-value was corrected using the Bonferroni method to adjust for multiple comparisons. The association between four psychological distress factors and UFs. was deemed statistically significant at a two-sided p-value below 0.0125 (α = 0.05/4 outcomes) and suggestive when the p-value was under 0.05. MR analyses were carried out using the TwoSampleMR (version 0.5.6) and MVMR (version 0.3) packages in R (version 4.3.1).

## Result

### Estimated causal effect of psychological distress on UFs

We selected 19, 45, 12, and 49 SNPs as genetic instruments for depressive symptoms, MDD, anxiety or panic attacks, and mood swings after LD clumping and removing pleiotropic SNPs, respectively (Supplementary Table S1-S4). The *F*-statistics for these genetic variants were above the critical value of 10, suggesting a minimal risk of weak instrumental bias.

**Fig. 2:**
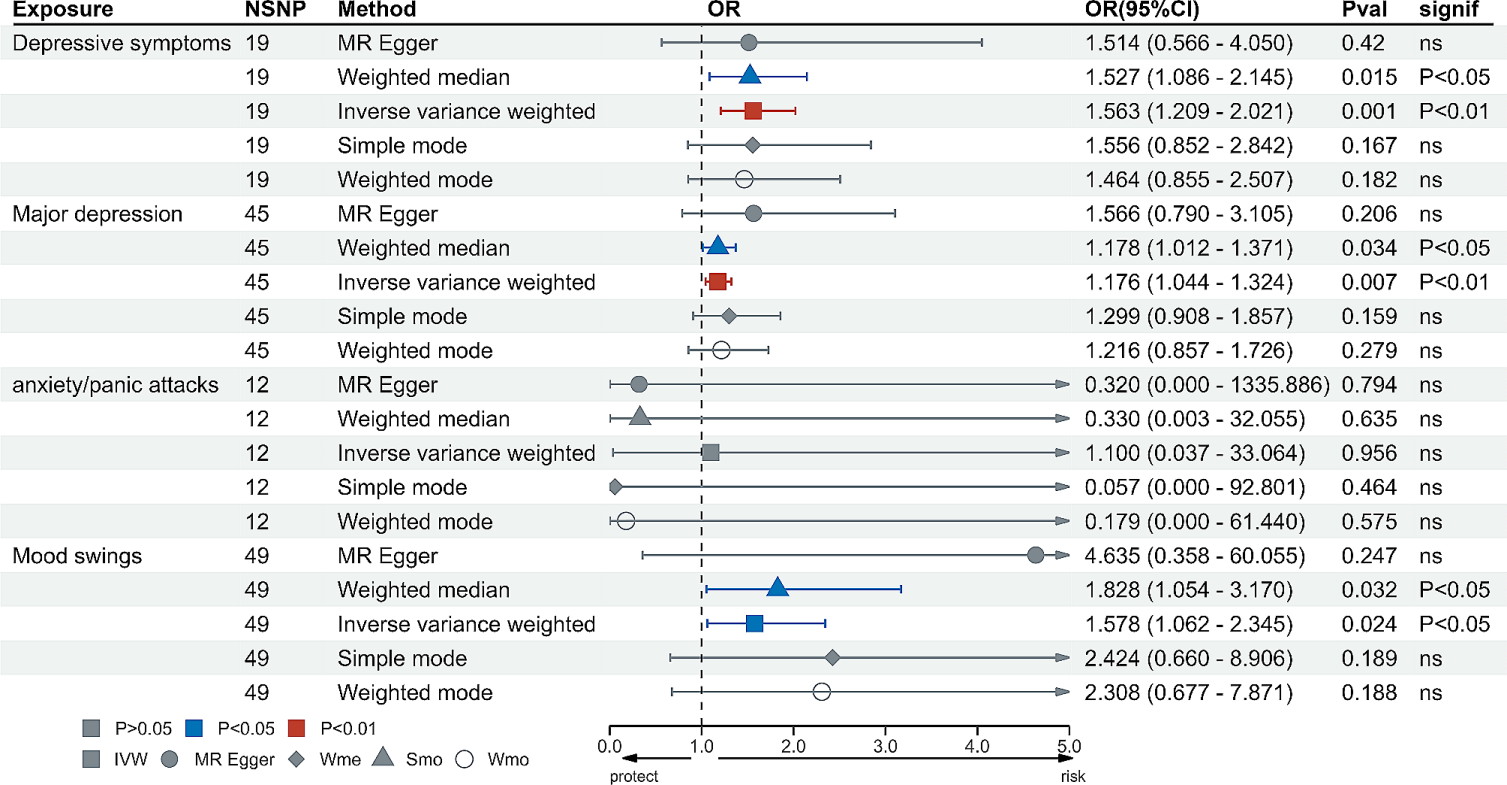
The effect of genetically determined psychological factors on UFs using UVMR.

Based on the application of the Bonferroni correction, the results of our UVMR analysis suggest that genetic predispositions to depressive symptoms (Odds Ratio [OR] = 1.563, 95% Confidence Interval [CI] = 1.209–2.021, *P* = 0.001) and MDD (OR = 1.176, 95% CI = 1.044–1.324, *P* = 0.007) are associated with an increased risk of UFs in the IVW model. Moreover, the IVW model showed a nominally significant positive correlation between mood swings (OR: 1.578; 95% CI: 1.062–2.345; *P* = 0.024) and UFs risk. The outcomes were consistent with the findings from the weighted median model. Nevertheless, the study could not identify any causal relationship between anxiety or panic attacks and UFs. Similar conclusions were drawn from the other four statistical models. Figure 2 demonstrates the causal link between genetic predictors of psychological distress and the risk of UFs.

**Fig. 3:**
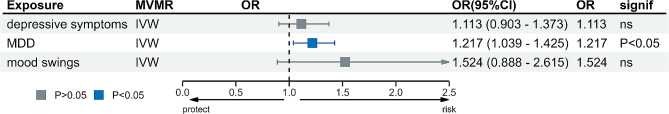
The direct effect of genetically determined psychological factors on UFs using MVMR-IVW controlled for BMI, smoking, alcohol intake frequency, and number of live births

Furthermore, after adjusting for confounders like BMI, smoking, alcohol intake frequency, and number of live births, the causal link between MDD and UFs remained significant (OR = 1.217, 95% CI = 1.039–1.425, *P* = 0.015). The causal relationship between depressive symptoms and UFs is no longer evident in the MVMR-IVW model (Fig. 3). The MVMR-Lasso and MVMR-Egger methods provided consistent results.

Notably, we found substantial proof for the direct effect of mood swings on UFs employing MVMR-Lasso (*P* = 0.028) and MVMR-Egger (*P* = 0.048) methods, while no association was found using the MVMR-IVW method (Table 2 ).

**Table 2:** The direct effect of genetically determined psychological factors on UFs using MVMR controlled for BMI, smoking, alcohol intake frequency, and number of live births

Exposures	MVMR	Beta	SE	*P*-value
depressive symptoms	MR-Lasso	0.043	0.094	0.650
	MR-median	0.148	0.124	0.235
MDD	MR-Lasso	0.195	0.063	0.002
	MR-median	0.209	0.087	0.017
mood swings	MR-Lasso	0.501	0.227	0.028
	MR-median	0.627	0.317	0.048

**Fig. 4:**
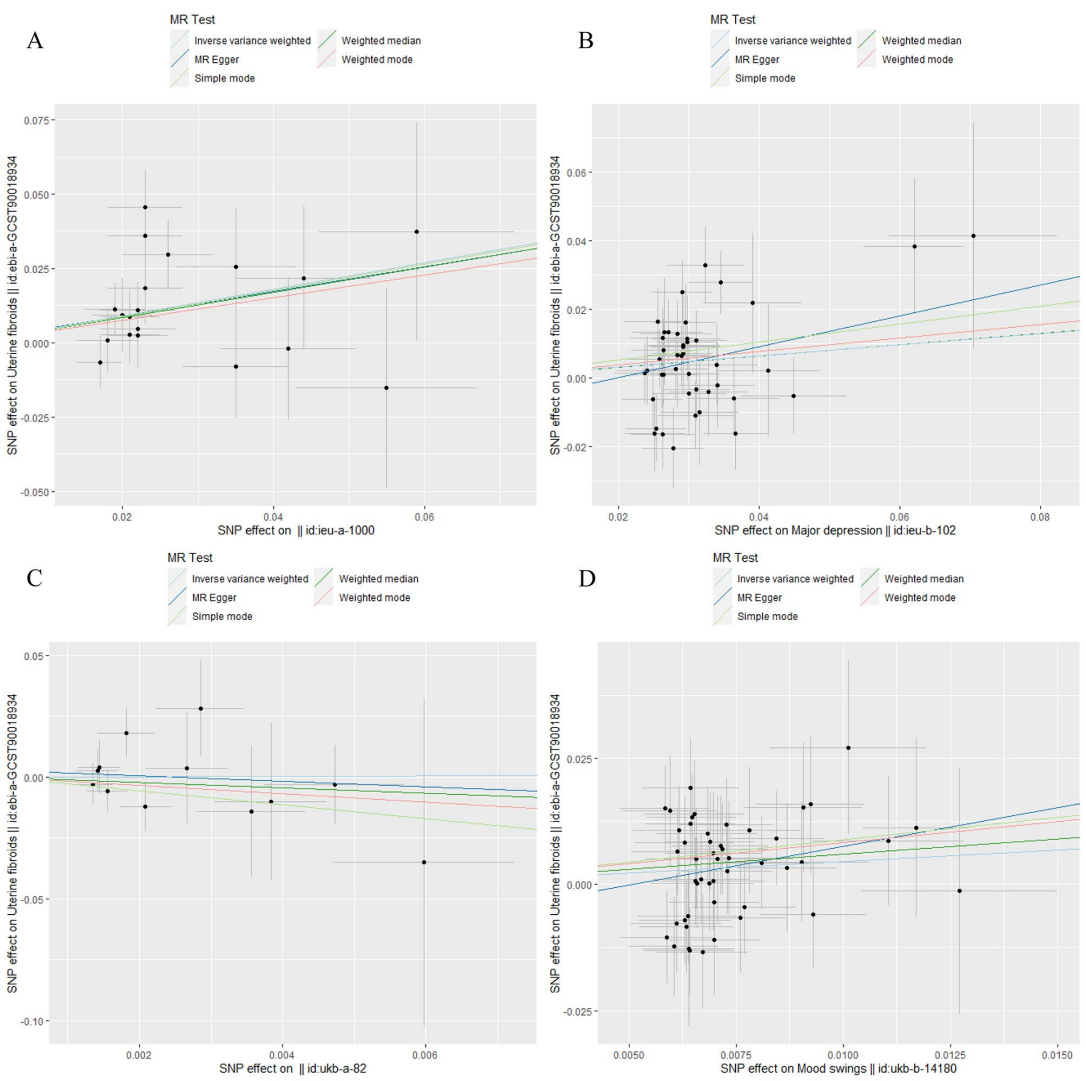
Scatter plots for MR analyses of the correlation between psychological factors and UFs in the IVW model. (A) depressive symptoms; (B) MDD; (C) anxiety or panic attacks; (D) mood swings

In the sensitivity analyses, MR-Egger regression results showed no evidence of directional pleiotropy. Cochran’s Q test results indicated an absence of heterogeneity. Additionally, the MR-PRESSO model identified no outliers. Table 3 provides detailed results from the sensitivity analyses. The scatter plots of the association between psychological distress factors and UFs are shown in Fig. 4. The leave-one-out plots suggest that particular SNPs are unlikely to influence the causal estimates (Supplementary Figure S1) significantly.

**Table 3:** Heterogeneity, horizontal pleiotropy, and MR-PRESSO tests of the associations between psychological factors and UFs

Exposures	Heterogeneity test			Pleiotropy test		MR-PRESSO
	method	Q-value	Pvalue	Egger-intercept	Q-value	Pvalue
depressive symptoms	IVW	22.364	0.216	0.0008	0.948	0.309
MDD	IVW	57.906	0.078	-0.009	0.409	0.073
anxiety or panic attacks	IVW	8.268	0.689	0.003	0.757	0.676
mood swings	IVW	38.754	0.827	-0.008	0.408	0.823

UFs, uterine fibroids; MDD, major depressive disorder; IVW, inverse variance weighted

### Estimated causal effect of UFs on psychological distress

We identified 34, 37, 37, and 37 SNPs as IVs for UFs to evaluate their associations with depressive symptoms, MDD, anxiety or panic attacks, and mood swings after LD clumping and removing pleiotropic SNPs, respectively, as detailed in Supplementary Tables S5-S8. All genetic variations taken into the reverse analysis possessed *F*-statistics that exceeded the essential threshold of 10, indicating a low risk of weak instrumental bias. Across all five statistical models, no causal relationship was found between genetic predisposition to UFs and depressive symptoms (OR: 1.012; 95% CI: 0.992–1.031, *P* = 0.245), MDD (OR: 0.996; 95% CI: 0.976–1.016, *P* = 0.673), anxiety or panic attacks (OR: 1.000; 95% CI: 0.998–1.001, *P* = 0.58), and mood swings (OR: 1.001; 95% CI: 0.996–1.005, *P* = 0.795). Supplementary Figure S2 in the paper illustrates the causal connections between the genetic prediction of UFs and these four emotional disorders. The sensitivity analysis revealed that the MR-Egger regression results did not indicate the presence of directional pleiotropy. The Cochran’s Q test yielded evidence of homogeneity. In addition, the MR-PRESSO model did not detect any outliers. Table 4 presents comprehensive findings derived from the sensitivity analyses. Supplementary Figure S3 displays the scatter plots illustrating the relation between UFs and psychological distress indicators. The leave-one-out plots indicate that specific SNPs are unlikely to impact the causal estimates majorly (Supplementary Figures S4).

**Table 4:** Heterogeneity, horizontal pleiotropy, and MR-PRESSO tests of the associations between UFs and psychological factors

Outcomes	Heterogeneity test			Pleiotropy test		MR-PRESSO
	method	Q-value	Pvalue	Egger-intercept	Pvalue	Pvalue
depressive symptoms	IVW	32.382	0.498	0.002	0.254	0.297
MDD	IVW	38.527	0.356	-0.002	0.423	0.305
anxiety or panic attacks	IVW	46.727	0.109	1.716e-06	0.990	0.167
mood swings	IVW	31.773	0.670	0.0004	0.399	0.602

UFs, uterine fibroids; MDD, major depressive disorder; IVW, inverse variance weighted

## Discussion

Our study employed the MR approach to examine the reciprocal causal association between four emotional disorders and UFs. Applying the Bonferroni adjustment to our results revealed a notable association between depressive symptoms, MDD, and the probability of having UFs. Similarly, there was a statistically significant association between mood swings and an elevated incidence of UFs. There is no genetic evidence to support the link between anxiety or panic attacks and UFs. The causal relationship between MDD and UFs remained statistically significant even after controlling for BMI, smoking, alcohol intake frequency, and number of live births. Nevertheless, in the MVMR model, the causality between depressive symptoms, mood swings, and UFs was not sustained. In addition, our reverse MR analysis did not yield genetic proof confirming a causal link between UFs and the four sorts of emotional disorders.

Our MR analysis demonstrated a positive connection between MDD and a heightened susceptibility to UFs, consistent with other research findings. An example of a prospective cohort study found that individuals who scored high on depression symptom ratings, as well as those who were diagnosed with depression by healthcare professionals, had an increased probability of developing UFs [9]. A recent meta-analysis has revealed that chronic psychological stress, similar to obesity and alcohol consumption, is a significant risk factor for the development of UFs (OR: 1.24; 95% CI: 1.15–1.34). Notably, chronic psychological stress is a primary contributor to MDD [30]. While our univariate MR analysis revealed an evident association between depressive symptoms, mood swings, and a raised incidence of UFs, this relation lost its significance after accounting for variables such as BMI, smoking, alcohol intake frequency, and number of live births. This suggests that the relationship between these two forms of psychological discomfort and UFs may be influenced by confounding factors. Another plausible explanation is that the associations between mental problems and UFs may vary depending on the severity of the condition. Even though mild depressive symptoms and mood swings may not increase the risk of UFs, MDD appears to be a genuine risk factor for UFs.

According to reports, women with UFs experience decreased quality of life (QOL) and mental health levels [31–34]. A cross-sectional observational study has indicated that women with UFs exhibit markedly elevated perceived stress scores compared to age-matched women of childbearing age, particularly those with significant menstrual bleeding [30]. Moreover, UFs are significantly associated with lower implantation rates, cumulative pregnancy, and live birth rates [35]. UFs can also increase the risk of adverse pregnancy outcomes [36], which severely impacts the mental health of women with fertility needs. There are research findings indicating that treatment for UFs improves anxiety and depression symptoms associated with symptomatic UFs [37]. However, definitive conclusions cannot be drawn regarding the potential benefits of surgical treatment for UFs on in vitro fertilization (IVF) outcomes, adverse maternal-fetal outcomes, and corresponding psychological states [36, 38, 39]. Additionally, a retrospective cohort study indicated an apparent rise in the likelihood of depression among individuals with UL compared to those without UL. However, it had certain limitations since it did not account for potential confounding variables such as reproductive history, smoking, and alcohol consumption [40]. Our MR study, in contrast, proved no correlation between UFs and four types of emotional disorders. This discrepancy between MR outcomes and observational study results might stem from the impact of confounding variables, indicating that the outcomes of prior observational studies may be compromised. For example, age over 40 years old is a risk factor for UFs [41], and issues such as decreased fertility caused by uterine aging with age are also confounding factors causing psychological distress in women [42]. Consequently, further investigation is warranted to explore the correlation between UFs and the tendency to emotional disorders.

While the precise mechanism linking severe depression to UFs remains unclear, several hypotheses are considered plausible. First, the psychological stress state associated with MDD can disrupt the functioning of the hypothalamic-pituitary-adrenal (HPA) and hypothalamic-pituitary-gonadal (HPG) axes. This disturbance affects the levels and bioavailability of steroid hormones such as estrogen and progesterone, which are essential in regulating uterine tissue growth and may directly promote the proliferation of uterine fibroid cells [43, 44]. Furthermore, the expression levels of neurotransmitters like norepinephrine (NE) are dysregulated in MDD patients. In vitro cell experiments have demonstrated that NE can regulate the levels of estrogen receptor (ER), progesterone receptor (PR), vascular endothelial growth factor (VEGF), and fibroblast growth factor (FGF) in human uterine leiomyoma cells. This regulation occurs through the AR-cAMP-PKA-dependent signaling pathway, potentially impacting the occurrence and development of fibroids [45]. Besides, individuals diagnosed with MDD had higher levels of nuclear factor kappa B (NF-κB) and pro-inflammatory cytokines, including interleukin-6 (IL-6) and interleukin-1β (IL-1β), when compared to the control group without depression [46]. . This elevation can promote fibrosis and angiogenesis in uterine leiomyoma cells by amplifying the inflammatory response. Such biological changes are conducive to the growth of leiomyomas and could contribute to the development of UFs [47].

Thirdly, there have been reports attaching depression to unhealthy lifestyle choices, including skipping breakfast, excessive consumption of processed foods, insufficient intake of protein and vitamins, prolonged sedentary behavior, and increased screen time [48, 49]. These lifestyle factors may play a role in the pathogenesis of UFs [50, 51]. Another potential mechanism involves the gut microbiota. Prior research has indicated a connection between depression and gut ecological dysbiosis, characterized by decreased fatty acid-degrading bacteria and short-chain fatty acids [52]. Dysfunctions in gut microbiota may heighten the likelihood of UFs by affecting immune-inflammatory responses and alterations in gut metabolites [53, 54]. Lastly, a recent study has discovered that depression and five reproductive endocrine illnesses, such as UFs and endometriosis, share a common genetic variant known as ESR1. This finding emphasizes the significant influence of estrogen signaling in the connection between emotional disorders and UFs [55]. However, research into the link between MDD and UFs remains limited, necessitating further investigation to elucidate the potential underlying mechanisms.

This is the first known instance where an MR Framework has been employed to assess the genetic relationship between psychological distress and UFs. This study has several strengths. Primarily, the MR approach minimizes confounding factors and reverse causation, providing robust evidence for causal relationships. Additionally, the substantial sample size from multiple GWAS bolsters our analysis’s statistical power and reliability, underpinning the established correlations. Moreover, the bidirectional analysis comprehensively explains the relationship between psychological distress and UFs. Furthermore, we performed an exhaustive sensitivity analysis to affirm the reliability of our findings. Finally, MVMR was employed to adjust for factors such as BMI, smoking, alcohol intake frequency, and number of live births, thereby elucidating the direct impact of emotional disorders and UFs. However, there are limitations to consider. Reliance on aggregated data from the GWAS database precludes evaluating the non-linear relationships between psychological distress and UFs. The inability to access sex-stratified data constrains deeper investigation into the more nuanced associations. Despite multiple sensitivity analyses, the potential for residual pleiotropy cannot be entirely ruled out. Lastly, the predominance of participants of European descent reduces demographic bias yet concurrently confines the broader applicability of our findings across diverse racial groups.

This study contributes to the existing literature by providing robust genetic evidence for the causal relationship between MDD and UFs. While previous observational studies have suggested a link between psychological distress and UFs, they are often limited by potential confounding factors and reverse causality. Our MR approach addresses these limitations. This study fills a crucial gap in understanding the etiology of UFs and underscores the importance of mental health in gynecological conditions. Our findings provide significant clinical implications for managing psychological distress in patients with UFs. Clinicians should consider incorporating psychological assessment and intervention into treatment plans to potentially reduce the incidence and severity of UFs.

Further research is necessary to explore the biological mechanisms underlying the relationship between psychological distress and UFs. Understanding these mechanisms could unveil new therapeutic targets and discover innovative treatment methods that address UFs’ psychological and biological aspects. Future research should include diverse racial groups to ensure the generalizability of the findings across different populations.

## Conclusion

Our MR study offers compelling evidence that a genetic predisposition to MDD is associated with an increased risk of UFs. Engaging in proactive assessment and intervention in women’s mental health could reduce the likelihood of developing UFs. Further investigation is necessary to understand better the biological pathways linking psychological distress and UFs. The results from UVMR and MVMR analyses provide limited evidence of a heightened likelihood of UFs due to depressive symptoms, emotional fluctuations, and anxiety or panic attacks. This suggests that the relationship between emotional disorders and UFs may depend on the severity of the disorder. Meanwhile, the outcomes of our reverse MR analysis did not yield genetic evidence for a causal relationship between UFs and emotional disorders. This finding implies that the connections observed in previous observational studies might be affected by confounding factors.

### Electronic supplementary material

Below is the link to the electronic supplementary material.

Supplementary Material 1

Supplementary Material 2

Supplementary Material 3

Supplementary Material 4

Supplementary Material 5

Supplementary Material 6

Supplementary Material 7

## Data Availability

The datasets for this study can be found in the IEU open GWAS project, at https://gwas.mrcieu.ac.uk/.
