# Supplementary figures and images for: Psychological distress and uterine fibroids: a bidirectional two-sample mendelian randomization study
Source: BMC Womens Health. 2024 Jun 18;24:351. doi: 10.1186/s12905-024-03196-8 (PMC11184690; doi:10.1186/s12905-024-03196-8)

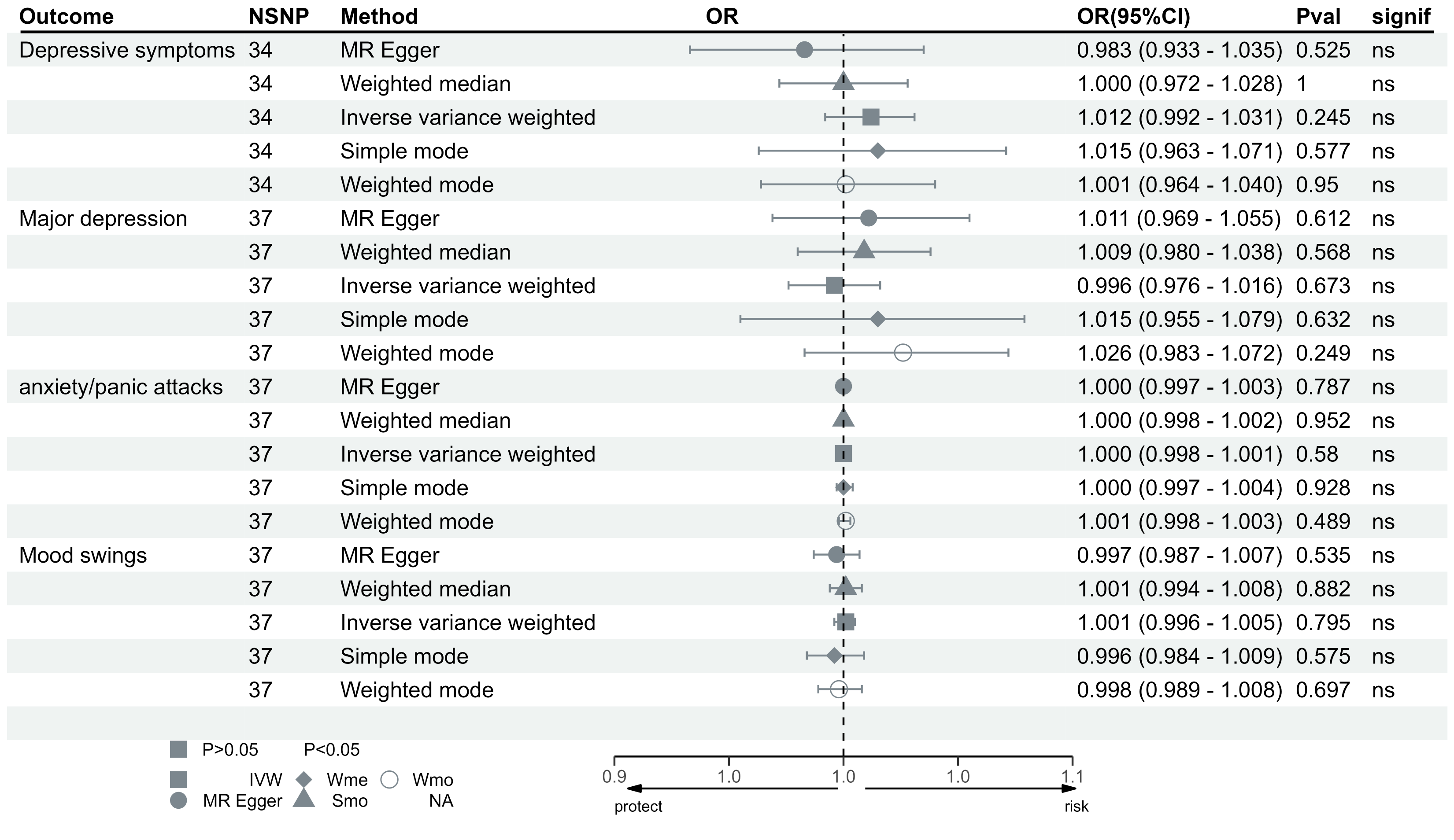

Supplement: Supplementary file 2 — Supplementary Material 2 [file 12905_2024_3196_MOESM2_ESM.tif]
