# Supplementary material for: Psychological distress and uterine fibroids: a bidirectional two-sample mendelian randomization study
Source: BMC Womens Health. 2024 Jun 18;24:351. doi: 10.1186/s12905-024-03196-8 (PMC11184690; doi:10.1186/s12905-024-03196-8)
Supplement: Supplementary file 7 — Supplementary Material 7 [file 12905_2024_3196_MOESM7_ESM.pdf]

Supplementary Figure S1 The leave-one-out plots for MR analyses of the correlation between psychological factors and UFs. (A) depressive symptoms; (B) MDD; (C) anxiety or panic attacks; (D) mood swings.

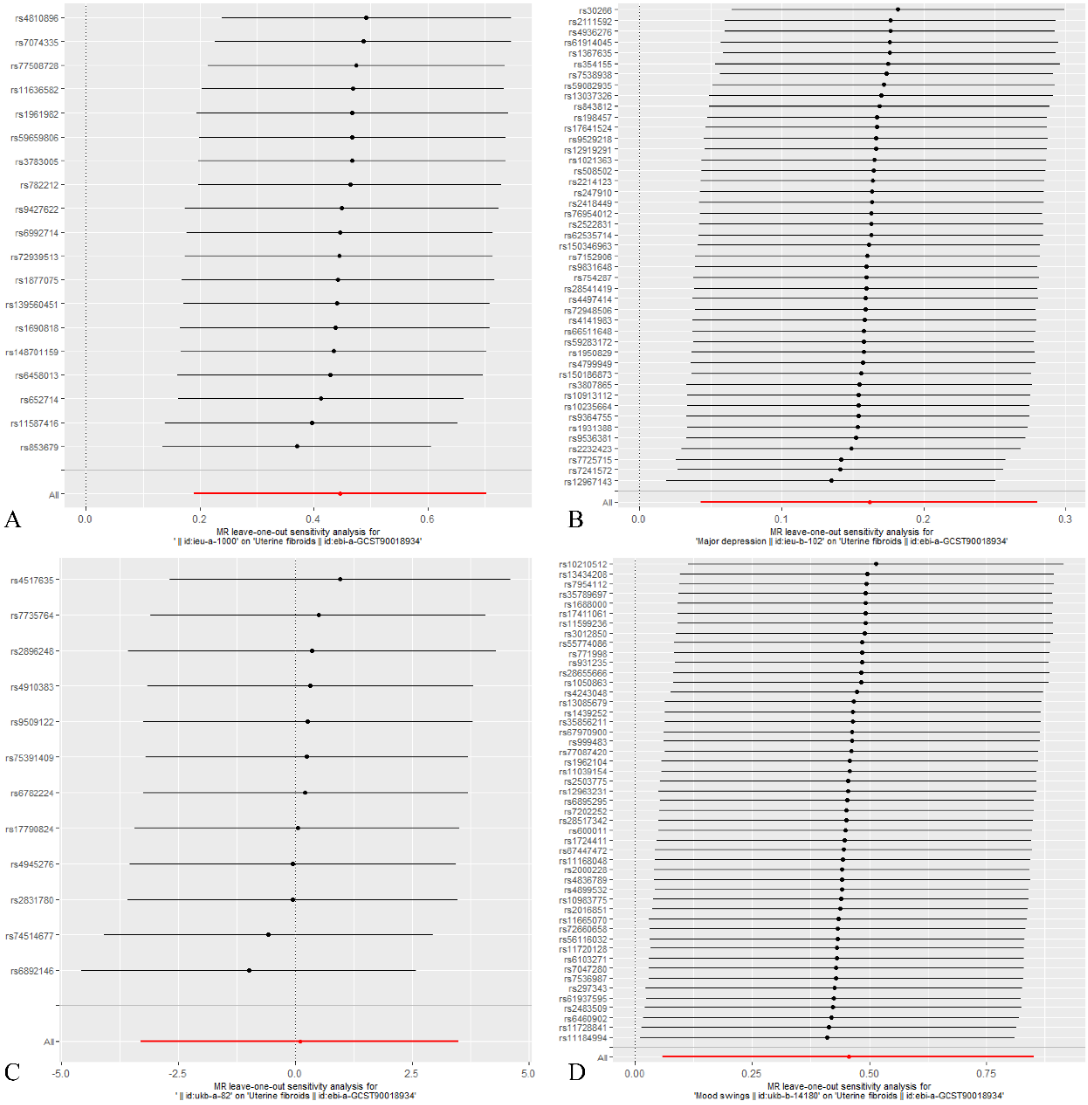

MR, mendelian randomization; UFs, uterine fibroids; MDD, major depressive disorder.

Supplementary Figure S2 The effect of genetically determined UFs on psychological factors using UVMR.

Outcome	NSNP	Method	OR(95%CI)	Pval	signif
Depressive symptoms	34	MR Egger	0.983 (0.933 - 1.035)	0.525	ns
	34	Weighted median	1.000 (0.972 - 1.028)	1	ns
	34	Inverse variance weighted	1.012 (0.992 - 1.031)	0.245	ns
	34	Simple mode	1.015 (0.963 - 1.071)	0.577	ns
	34	Weighted mode	1.001 (0.964 - 1.040)	0.95	ns
Major depression	37	MR Egger	1.011 (0.969 - 1.055)	0.612	ns
	37	Weighted median	1.009 (0.980 - 1.038)	0.568	ns
	37	Inverse variance weighted	0.996 (0.976 - 1.016)	0.673	ns
	37	Simple mode	1.015 (0.955 - 1.079)	0.632	ns
	37	Weighted mode	1.026 (0.983 - 1.072)	0.249	ns
anxiety/panic attacks	37	MR Egger	1.000 (0.997 - 1.003)	0.787	ns
	37	Weighted median	1.000 (0.998 - 1.002)	0.952	ns
	37	Inverse variance weighted	1.000 (0.998 - 1.001)	0.58	ns
	37	Simple mode	1.000 (0.997 - 1.004)	0.928	ns
	37	Weighted mode	1.001 (0.998 - 1.003)	0.489	ns
Mood swings	37	MR Egger	0.997 (0.987 - 1.007)	0.535	ns
	37	Weighted median	1.001 (0.994 - 1.008)	0.882	ns
	37	Inverse variance weighted	1.001 (0.996 - 1.005)	0.795	ns
	37	Simple mode	0.996 (0.984 - 1.009)	0.575	ns
	37	Weighted mode	0.998 (0.989 - 1.008)	0.697	ns

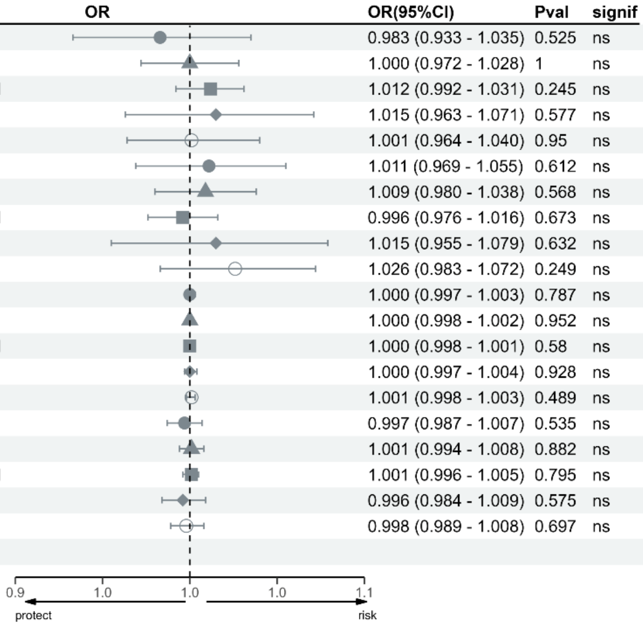

P>0.05 P<0.05
IVW Wme Wmo
MR Egger Smo NA

UFs, uterine fibroids; UVMR, univariate mendelian randomization.

Supplementary Figure S3 The scatter plots for MR analyses of the correlation between UFs and psychological factors in the IVW model. (A) depressive symptoms; (B) MDD; (C) anxiety or panic attacks; (D) mood swings.

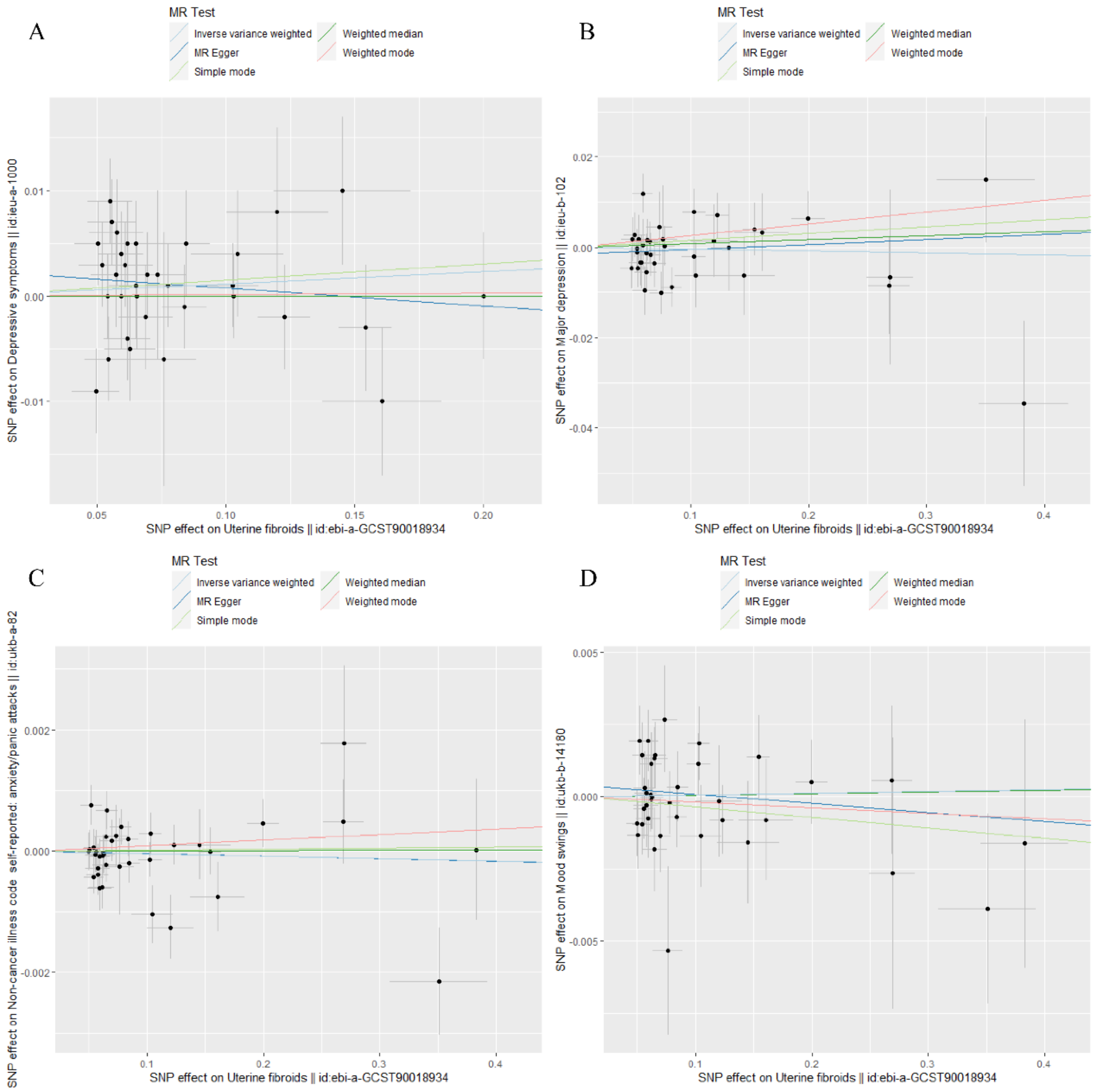

MR, mendelian randomization; UFs, uterine fibroids; IVW, inverse variance weighted; MDD, major depressive disorder.

Supplementary Figure S4 The leave-one-out plots for MR analyses of the correlation between UFs and psychological factors. (A) depressive symptoms; (B) MDD; (C) anxiety or panic attacks; (D) mood swings.

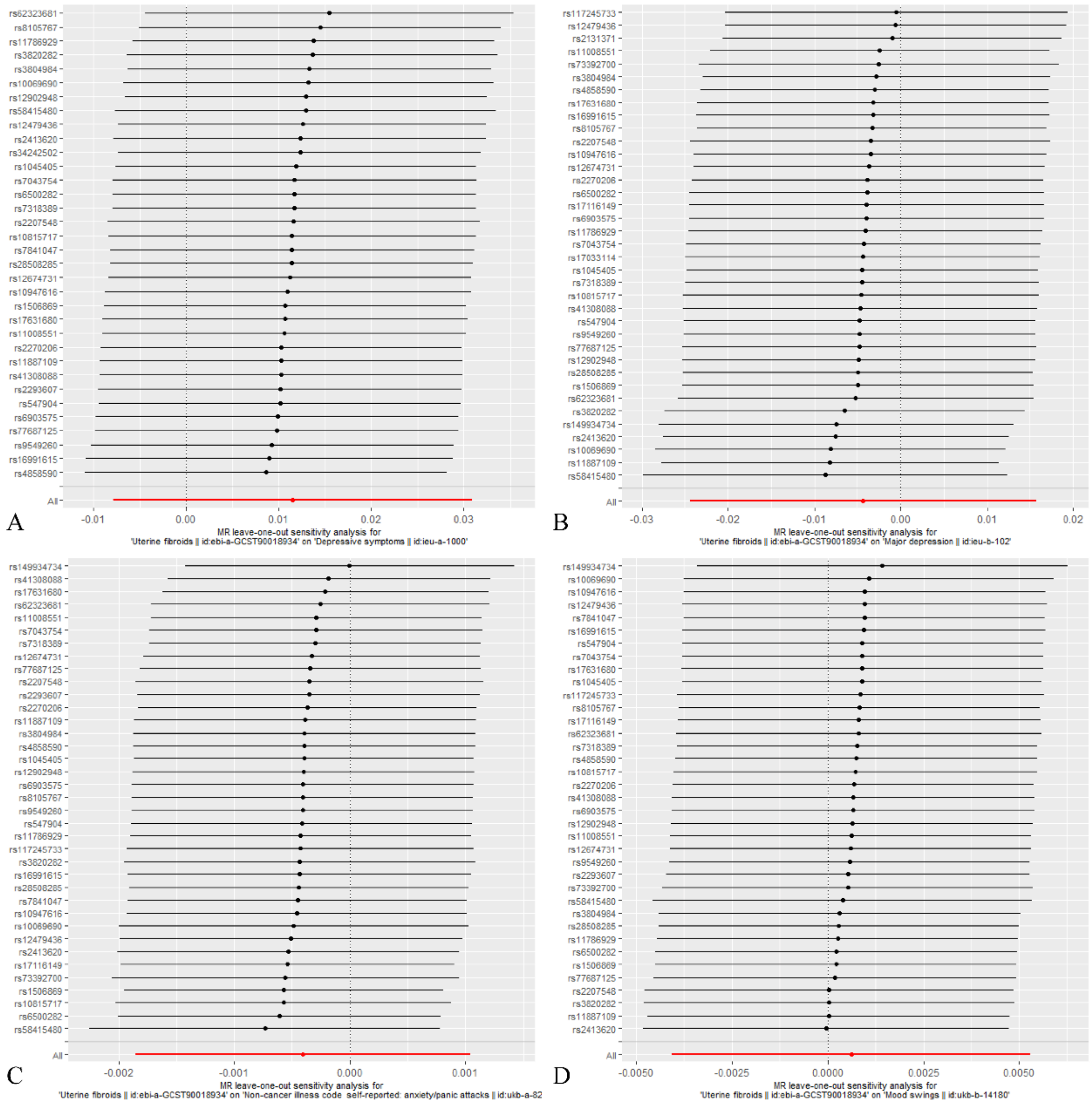

MR, mendelian randomization; UFs, uterine fibroids; MDD, major depressive disorder.
